# Air-stable, long-length, solution-based graphene nanoribbons†

**DOI:** 10.1039/d0sc02105a

**Published:** 2020-09-09

**Authors:** Samuel R. Peurifoy, Qizhi Xu, Richard May, Natalia A. Gadjieva, Thomas J. Sisto, Zexin Jin, Lauren E. Marbella, Colin Nuckolls

**Affiliations:** Department of Chemistry, Columbia University New York New York 10027 USA cn37@columbia.edu; Department of Chemical Engineering, Columbia University New York New York 10027 USA; XL Batteries 760 Parkside Avenue Brooklyn NY 11226 USA

## Abstract

Within the context of nanoelectronics, general strategies for the development of electronically tunable and air stable graphene nanoribbons are crucial. Previous studies towards the goal of processable nanoribbons have been complicated by ambient condition instability, insolubility arising from aggregation, or poor cyclization yield due to electron deficiency. Herein, we present a general strategy for the elongation of smaller graphene nanoribbon fragments into air-stable, easily processed, and electronically tunable nanoribbons. This strategy is facilitated by the incorporation of electron-rich donor units between electron-poor acceptor perylene diimide oligomeric units. The ribbons are processed in solution *via* a visible-light flow photocyclization using LEDs. The resulting long nanoribbons can be solution-cast and imaged, which are necessary characteristics for device fabrication. The ribbons become conductive after thermolysis of the pendent side-chains. The electron-accepting character of these nanoribbons in solution is reversible, and the conductivity of the thermolyzed species as a solid remains stable. This work highlights our general strategy for the mild and reliable fabrication of tunable and ambient-stable graphene nanoribbons, and charts a straightforward route for facile device incorporation.

## Abstract 2

A strategy is shown for the elongation of graphene nanoribbon (GNR) fragments into air-stable, solution processable and electronically tunable GNRs, aided by incorporating electron-rich donor units between electron-poor oligomeric acceptor units.

## Graphical abstract

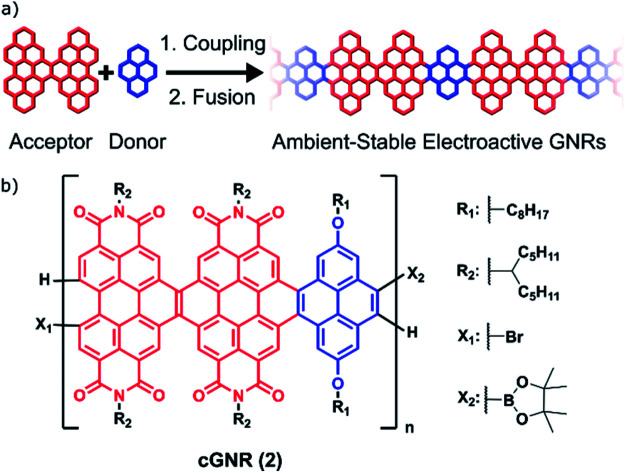

## Introduction

This manuscript describes a facile method for creating long, air-stable, electronically functionalized nanoribbons that can be easily processed from solution and, once deposited, can be triggered to become conductive in thin films. Graphene nanoribbons (GNRs) are promising candidates as device components in nanoscale electronics.^1–9^ The state of the art for GNR implementation in the context of nanoelectronics centers on their surface-mediated synthesis and characterization.^1–4^ These surface-mediated approaches benefit from well-understood radical coupling chemistry and an increasing number of methods to introduce both dopant sites^10–12^ and interesting topological states.^13–15^ However, methods using the surface as a synthetic medium suffer both from drawbacks in ambient stability and processing-related difficulties of removing the GNRs from a surface.^16,17^ Furthermore, because the surface-mediated pathway generally produces insoluble GNRs arrayed randomly across a surface, the precise positioning and placement of these surface-synthesized nanoribbons on arbitrary devices is difficult.^17^

**Fig. 1:**
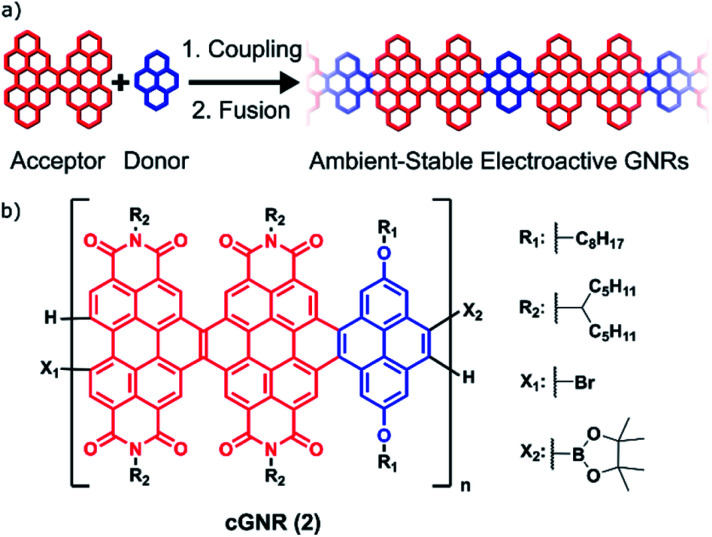
(a) A general strategy for synthesizing extended solution-processed graphene nanoribbons. This strategy builds off of a previously successful strategy of incorporating electron-rich linkers between electron-deficient ribbons.^20^ (b) Polymer subunit described herein, after coupling and fusion. The polymer is generated from a regioisomeric mixture of dimeric perylene diimide acceptors (red) and pyrene donors (blue), which converge upon cyclization.

Solution-based syntheses have the capacity to overcome some of the problems associated with surface-initiated synthesis while simultaneously creating atomically precise and electronically tunable nanoribbons. However, the majority of reported solution-based syntheses rely on harsh chemical conditions (Scholl reactions) to achieve the key aromatization reactions, which can create defects in the GNRs.^17^ Some milder methods have been developed (APEX), but usually are not amenable to the incorporation of electronically tunable components.^18^ To address this, we now demonstrate the synthesis of long, electronically tunable, and ambient-stable nanoribbons formed by the mild copolymerization and fusion of π-electron-poor perylene diimide (PDI) oligomers^7^ with π-electron-rich pyrene subunits (Fig. 1a).

Crucially, the Suzuki reactions necessary to couple them together into polymers and the subsequent photocyclization reactions to fuse the polymers into fully conjugated ribbons are both quantitative and utilize mild methods.^19–21^ This two-step method of coupling followed by fusion permits the rapid synthesis of semiconductive contorted n-type GNRs that have lengths of ∼50 nm (**cGNR**; Fig. 1b). These largely defect-free and selectively functionalized contorted GNRs are soluble, ambient stable, and easily imaged as lamellar aggregates when cast into thin films.

## Results and discussion

**Scheme 1:**
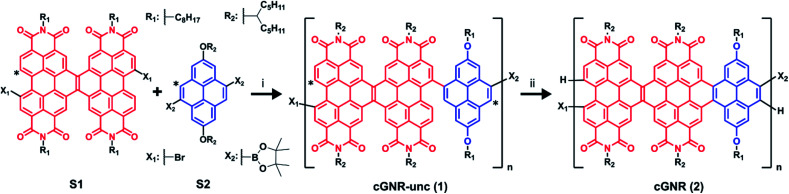
Synthesis of PDI-based GNRs. Key: (i) 1 eq. **hPDI2-Br2** (**S1**), 1 eq. **diborylated pyrene** (**S2**), 8 eq. K_2_CO_3_, 0.01 eq. **Pd(dppf)Cl2**, 0.1 M THF/H_2_O (5 : 1). Air-free conditions, 57 °C, 12 h. (ii) 1 eq. **cGNR-unc**, 5 eq. I_2_, 0.05 M chlorobenzene. Visible-light flow reactor, 48 h. Asterisks indicate alternate sites of X_1_, X_2_ functional groups, as both precursors are synthesized in regioisomeric mixtures.

Scheme 1 details the synthesis of the polymeric nanoribbons. Suzuki coupling between a diborylated linker^20^ and a dihalogenated PDI subunit^7^ (**S1** and **S2**) provides a polymer (**cGNR-unc**; **1**) wherein the dimeric PDI moieties are bridged with a pyrene subunit. After an aqueous wash of the reaction mixture followed by consecutive methanol and hexanes Soxhlet extractions to remove starting materials, we subject uncyclized **1** to a visible light flow photocyclization/oxidation to yield cyclized **2** (see ESI† for synthetic procedure details). Then, we separate the product into dichloromethane-soluble (DCM) and chloroform-soluble (CHCl_3_) fractions. The DCM fraction contains polymers of moderate length and is 36% of final yield. The CHCl_3_ soluble fraction contains longer-length polymers and is 64% of final yield.

Gel permeation chromatography (GPC) calibrated to polystyrene size-exclusion chromatography (SEC) standards^22^ reveals that the moderate-length fraction exhibits *M*_n_ = 7908, *M*_w_ = 18 998, and PDI = 2.4, and that the long-length fraction exhibits *M*_n_ = 30 213, *M*_w_ = 66 689, and PDI = 2.2 (Fig. S3†). From the GPC-derived molecular weights we can estimate the number of subunits in each of these two fractions,^23^ and obtain a crude estimate of the length of the GNRs. Using this method, we estimate that the moderate-length GNRs are ∼25 nm in length and that the long-length GNRs are ∼50 nm in length. MALDI-TOF analysis^18^ proved to be prohibitively difficult, but spectra are provided in the ESI (Fig. S4†). Note that the choice of a Suzuki polymerization over a Diels–Alder cycloaddition polymerization limits the molecular weight, as Suzuki couplings can suffer from side reactions that can prematurely halt the synthetic process.

**Fig. 2:**
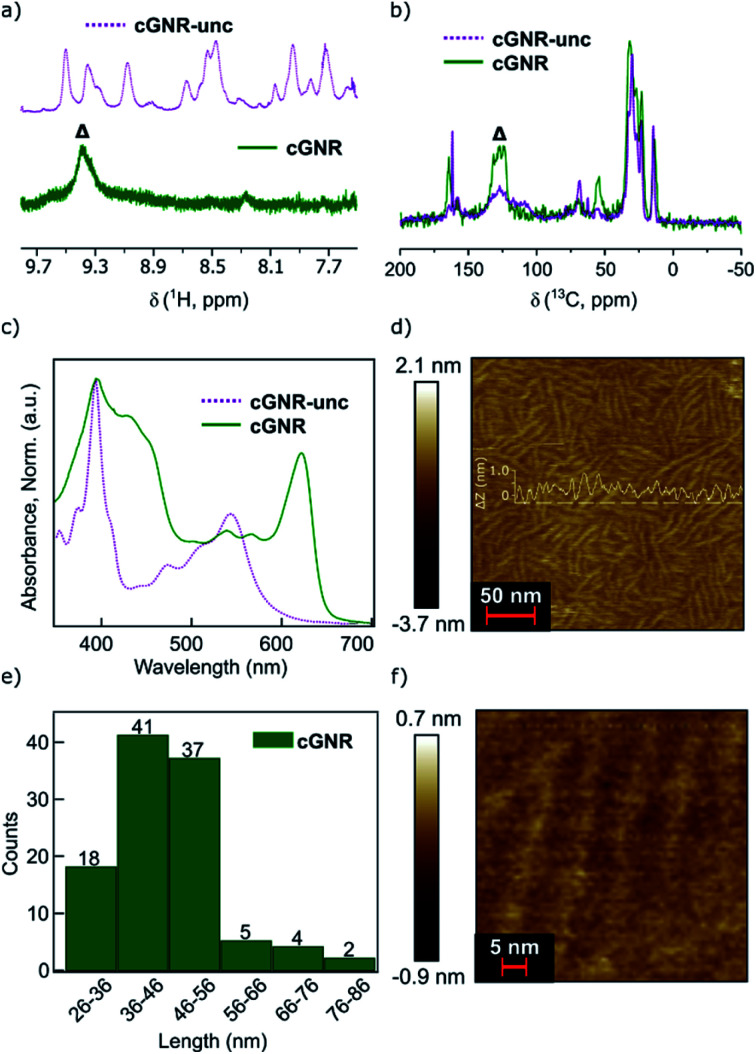
(a) ^1^H NMR overlay of uncyclized **1** (top) and cyclized **2** (bottom). Note the disappearance of disordered peaks associated with uncyclized positions, and the appearance of a consolidated peak associated with cyclization (marked by Δ).^20^ (b) ^1^H–^13^C CPMAS NMR overlay of uncyclized **1** (purple) and cyclized **2** (green). Note the convergence of the broad signals in the aromatic region, indicative of cyclization (marked by Δ). (c) Electronic absorption spectra for uncyclized **1** and cyclized **2** in the solution state. Note the redshift from resulting from photocyclization. (d) AFM map of cyclized **2** cast onto HOPG. Average ribbon length visible is approximately 50 nm. White overlay displays height profile of the monolayer, given in Δ*Z* (nm). (e) Histogram of ribbon lengths in (d). (f) AFM map of cyclized **2** cast onto HOPG, magnified to 5 nm resolution.

Solution (^1^H; Fig. 2a) and solid-state (^13^C; Fig. 2b) NMR confirm the reaction proceeds to completion, providing nearly defect-free ribbons. The numerous aromatic ^1^H resonances in the uncyclized product converge to a singular, yet broad, aromatic resonance upon cyclization, which likely also represents a chemical shift distribution. Likewise, the ^1^H–^13^C cross-polarization magic-angle spinning (CPMAS) solid-state NMR spectrum of the uncyclized product shows a large distribution of aromatic environments. Upon cyclization, the aromatic environments become more uniform, which is consistent with the narrowing of the corresponding ^13^C aromatic resonances now centered at approximately 128 ppm.^24^ We have reported similar transformations for the fusion of other large PDI-based systems.^19–21^

In comparing the electronic spectra of uncyclized **1** and cyclized **2** (Fig. 2c), the shift in *λ*_max_ and broadened absorptivity further support reaction completion.^7^ Similar to reported single molecule ribbons,^20^ the absorption maxima of cyclized **2** occurs around 624 nm. This bathochromic shift from a *λ*_max_ of 542 nm in uncyclized **1** to 624 nm in cyclized **2** is a result of extending the effective conjugation length along the nanoribbon axis by photocyclization. Solid-state electronic absorption spectra are provided in the ESI (Fig. S5†).

We can directly image the ribbons using atomic force microscopy (AFM), similar to other previously prepared GNRs.^16,18^ We created a sample for AFM imaging by spin-coating the long-length fractions of cyclized **2** onto highly-ordered pyrolytic graphite (HOPG). Details for the film formation conditions can be found in the ESI.† From the micrograph in Fig. 2d, the graphene nanoribbon lengths are distributed about 40–50 nm (Fig. 2e). The lengths of the nanoribbons determined by microscopy are similar to the lengths observed using the GPC approximation method described above. The width of each of these ribbons measured by AFM is around ∼3.8 nm, while the width of the nanoribbon including the side-chains measured by molecular modeling is ∼2.5 nm. For ease of comparison, we have overlaid a molecular model in the image Fig. 2f. The difference between the calculated and measured width of the ribbons arises from error native to the AFM tip radius.^25^

**Fig. 3:**
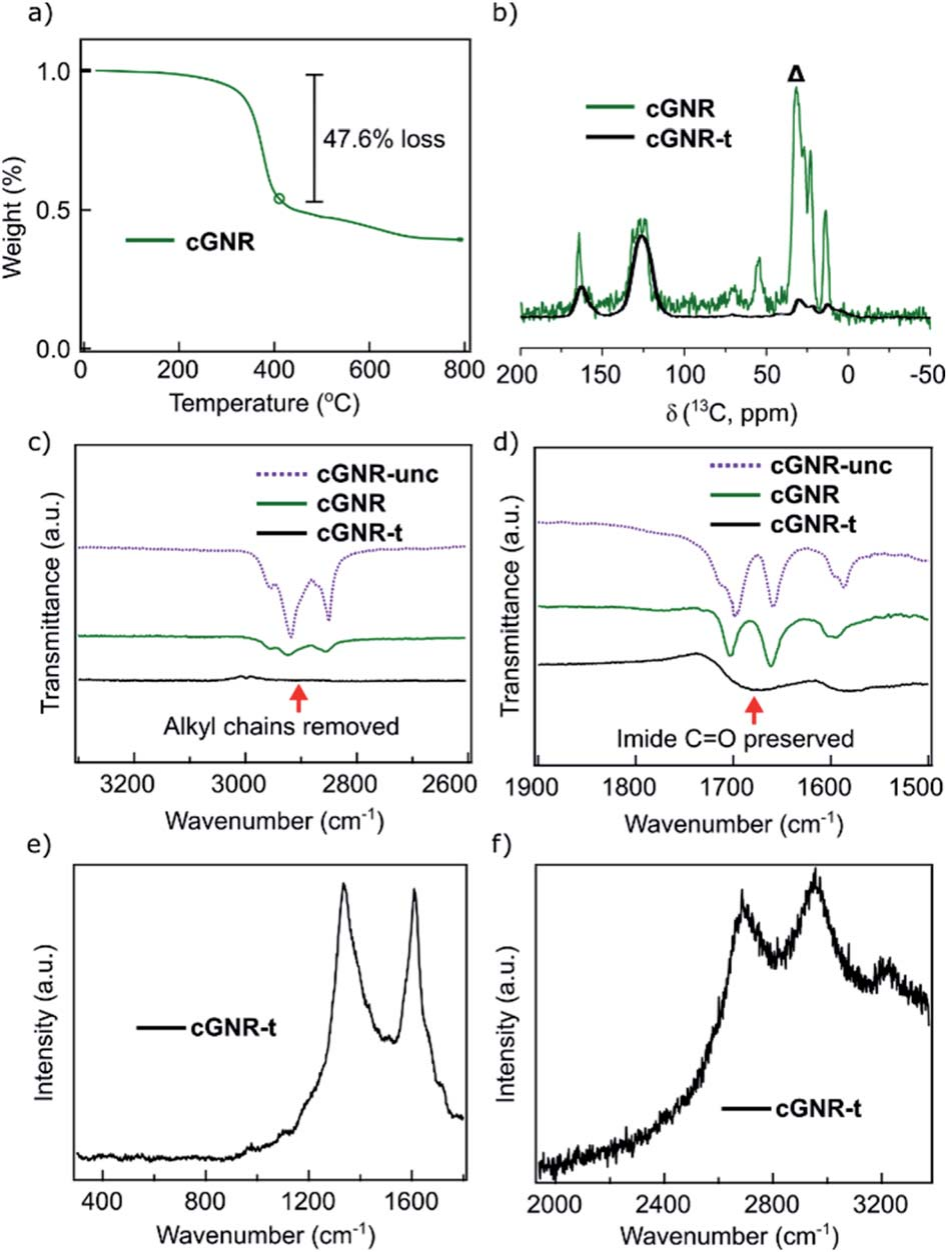
(a) TGA trace of cyclized **2** under a constant stream of N_2_. ∼47% loss observed, which we attribute to the removal of alkyl moieties (∼43% predicted). Calculation point highlighted with a circle. (b) ^1^H–^13^C CPMAS solid-state NMR showing the disappearance of the aliphatic carbons. (c) Fourier-transform infrared spectroscopy (IR) traces of the alkyl region (∼2900 cm^−1^). Note the disappearance of the alkyl signal following thermolysis from cyclized **2** to thermolyzed **3**. (d) IR traces of the imide region (∼1680 cm^−1^). Note the preservation of the imide C=O signal following thermolysis. (e) Raman spectroscopy trace for thermolyzed **3**. Note the presence of sharp D-band (∼1334 cm^−1^) and G-band (∼1608 cm^−1^) peaks, indicating the graphene nanoribbon architecture has been preserved through the thermolysis process. (f) Raman spectroscopy trace for thermolyzed **3**. Note the presence of the 2D (2686 cm^−1^), D + G (2955 cm^−1^), and 2G (3230 cm^−1^) features, again indicating the preservation of the graphene nanoribbon architecture.

There is a synergistic solubilizing effect from both the contortion along the nanoribbon backbone and the alkyl groups that adorn the imides, together providing high solubility and processability.^7^ This is in contradistinction to surface-synthesized GNRs that are difficult to process and precisely incorporate into devices.^17^ For our solution synthesized ribbons reported herein, the alkyl sidechains insulate the GNR core much in the same way that a plastic wrapping insulates macroscopic wires. Importantly, we have previously shown that the branched imide sidechains can be removed under vacuum thermolysis conditions.^26^ The thermogravimetric (TGA) trace of **2** shows an abrupt mass loss (∼47%; Fig. 3a) at 380 °C that approximately corresponds to the mass percentage loss predicted upon removal of the alkyl chains (∼43% predicted).^26^

We refer to this thermolyzed polymer as **cGNR-t** (**3**). Using a vacuum-sealed glass tube, when cyclized **2** is held at 400 °C for two hours, it is converted into thermolyzed **3** along with a clear, oily condensate, which is visible at the cool end of the tube. GC-MS analysis of the oily material confirms it is undecene, which is the elimination product from the branched imide sidechain (Fig. S6†). We see no GC-MS evidence of octane derivatives that could form from the ether-substituted pyrene linker, which may be due to the volatility of the octane derivatives formed. Solid-state NMR reveals near-quantitative removal of alkyl moieties, and retention of characteristic peaks in the aromatic and carbonyl regions (Fig. 3b). IR analysis (Fig. 3c and d) of the thermolyzed product further underscores that all alkyl groups are removed but the imides are retained (for fingerprint region, see Fig. S7†).^26^ This thermolysis step is a significant improvement over previous reports of similar methods on other GNR systems, which managed only modest alkyl thermolysis^25^ and unpredictable edge character.

Raman spectroscopy on the thermolyzed polymer samples (powder, 532 nm excitation wavelength) further evidences the formation of intact and alkyl-free GNRs. The spectra (Fig. 3e and f) of thermolyzed **3** display the first order D-band and G-band at 1334 cm^−1^ and 1608 cm^−1^ respectively that are consistent with reported values for fully cyclized GNRs synthesized from bottom-up^27^ and top-down methods.^28,29^ Well-resolved double-resonant signals were also observed at 2686 cm^−1^, 2955 cm^−1^ and 3230 cm^−1^, corresponding to the 2D, D + G, 2G bands, respectively. The Raman spectrum of unthermolyzed **2** also displays the D and G bands, however, the strong fluorescence background prevented us from obtaining a well-resolved spectrum (Fig. S8b†).

We do not observe these Raman transitions in the spectrum of uncyclized **1** (Fig. S8c†), which corroborates the idea that long-range cyclization is the foundation of GNR-attributed Raman character. These results underscore the successful long-range cyclization of the material into a well-formed GNR architecture, as well as the survival of the GNR architecture following thermolysis.

**Fig. 4:**
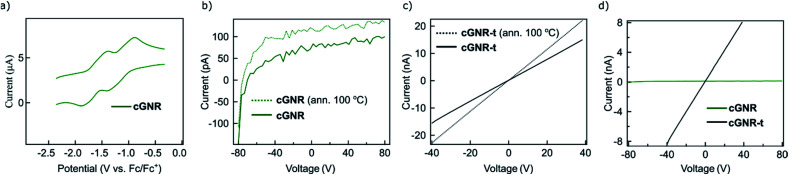
(a) Cyclic voltammogram (CV) of cyclized **2**, displaying reversible electron-accepting behavior. (b) Pellet current of cyclized **2** plotted as a function of drain voltage (*V*_D_), displaying poor conductance at all drain voltages. Dotted trace is annealed at 100 °C. (c) Pellet current of thermolyzed **3** plotted as a function of drain voltage (*V*_D_), displaying enhanced conductance after thermolysis. Dotted trace is annealed at 100 °C. (d) Comparison of pellet currents of both cyclized **2** and thermolyzed **3** plotted as a function of drain voltage. Thermolysis of the nanoribbons permits access to conductive GNRs synthesized off-surface.

The electronic tunability of the system described above is a key benefit to solution-based GNR fabrication. Our chosen architecture exhibits reversible electron-accepting behavior (Fig. 4a), which is necessary for the ultimate fabrication of semiconductive nanoelectronics. To evaluate the bulk characteristics, we pressed pellets from both cyclized **2** and thermolyzed **3** nanoribbons and performed two-terminal current–voltage curve measurements of the resistances by using silver paste as contacts. The thermolyzed (400 °C, 2 hours) variant **3** displays an increase in its electrical conductivity, exhibiting a conductivity of ∼2.3 μS m^−1^.

The conductivity for thermolyzed **3** is similar to that measured in a bulk conductivity study of unfunctionalized chevron GNRs.^30^ The comparison of the *I*–*V* curves for cyclized **2** (Fig. 4b) and thermolyzed **3** (Fig. 4c) clearly demonstrate an enormous increase in the current upon thermolysis (Fig. 4d); the conductivity of **3** is at least two orders of magnitude higher due to the removal of the insulating alkyl chains. This finding implies that the fabrication strategy outlined above produces GNRs that maintain ambient stability and electronic functionality in the bulk, and can be reliably triggered to become conductive *via* simple thermal processing.

## Conclusion

In conclusion, this study describes a facile and reliable method for the construction of ambient-stable and electronically tunable solution-processed GNRs. These contorted GNRs exhibit conductivity upon thermal activation, and can be coerced into lamellar aggregates on-surface for ease of device processing. The electronic character of these longer graphene nanoribbons can be easily tuned *via* the rich chemistry of the PDI subunit, or by a potential host of similar graphene fragment candidates. Such a reaction strategy, in which solution-processed, air-stable, and electronically active GNRs are thermolyzed to permit enhanced conductivity, is easily generalizable to new linker groups. This paper highlights the simplicity and robustness of the approach, and readily inspires numerous similar material architectures for advanced nanoelectronics fabrication.

## Conflicts of interest

There are no conflicts to declare.

## Supplementary Material

SC-011-D0SC02105A-s001
