# Supplementary material for: Air-stable, long-length, solution-based graphene nanoribbons
Source: Chem Sci. 2020 Sep 9;11(36):9978–82. doi: 10.1039/d0sc02105a (PMC8162120; doi:10.1039/d0sc02105a)
Supplement: SC-011-D0SC02105A-s001 [file SC-011-D0SC02105A-s001.pdf]

Electronic Supplementary Material (ESI) for Chemical Science.
This journal is © The Royal Society of Chemistry 2020

Air-Stable, Long-Length, Solution-Based Graphene Nanoribbons

Samuel R. Peurifoy,[a] Qizhi Xu,[a] Richard May,[b] Natalia A. Gadjieva,[a] Thomas J. Sisto,[c] , Zexin Jin,[a] Lauren E. Marbella,[b] Colin Nuckolls*,[a]

[a.] *Department of Chemistry, Columbia University, New York, New York 10027, United States.*
[b.] *Department of Chemical Engineering, Columbia University, New York, New York 10027, United States.*
[c.] *XL Batteries, 760 Parkside Avenue, Brooklyn, NY 11226, United States.*

*cn37@columbia.edu

Table of Contents

1) **General Experimental, Materials, Instrumentation** S2
2) **Procedures and Characterization** S2
   a. **cGNR-unc (1)** S3
   b. **cGNR (2)** S4
3) **^{1}H NMR Spectra** S5
4) **Gel permeation chromatography (GPC)** S7
5) **MALDI-TOF** S7
6) **Electronic absorption (UV-vis)** S7
7) **Gas chromatography-mass spectrometry (GC-MS)** S8
8) **IR Fingerprint Region** S8
9) **Raman spectroscopy** S8
10) **Additional doping experiments** S9
11) **References** S10

General Experimental Details: All reactions were performed in oven-dried or flame-dried round bottom flasks, unless otherwise noted. Such flasks were fitted with rubber septa and reactions were conducted under a positive pressure of nitrogen. Anhydrous and anaerobic solvents were obtained from a Schlenk manifold with purification columns packed with activated alumina and supported copper catalyst (Glass Contour, Irvine, CA).

Materials: hPDI2-Br_2 and diborylated-dioctyloxo-pyrene were synthesized according to our reported procedures.[1] All chemical reagents were purchased from commercial sources and used without purification unless noted.

Liquid NMR: Solution-state ^{1}H and ^{13}C NMR spectra were recorded on a Bruker DMX500 (500 MHz) spectrometer. Chemical shifts for protons are reported in parts per million downfield from tetramethylsilane and are referenced to residual protium within the NMR solvent ($CHCl_3$: δ 7.26). Chemical shifts for carbon are reported in parts per million downfield from tetramethylsilane and are referenced to the carbon resonances of the solvent ($CDCl_3$: δ 77.0). Data are represented as follows: chemical shift, multiplicity, (s = singlet, d = doublet, t = triplet, m = multiplet, b = broad, bm = broad multiplet), coupling constants in hertz, and integration.

SSNMR: Solid-state NMR (SSNMR) experiments were performed on a Bruker Avance NEO 600 MHz spectrometer equipped with a 1.6 mm HFXY MAS (magic-angle spinning) Phoenix NMR probehead operating at 599.37 MHz for ^{1}H and 150.71 MHz for ^{13}C. Samples were packed in 1.6 mm ZrO_2 rotors and sealed with Torlon caps for measurement. Due to limitations in sample quantity, certain samples were diluted with KBr to fill the rotor (2:1 KBr:high MW uncyclized **cGNR-unc** w:w, 2:1 KBr:rPDI-2, 6:1 KBr:uncyclized **hPDI2-Pyr-hPDI2**, 6:1 KBr:low MW uncyclized **cGNR-unc**). These differences in sample quantity are likely responsible for the differences in signal-to-noise observed between samples (e.g. Figure 3b, main text). Prior to each set of experiments, the magic-angle was set using KBr. ^{1}H-^{13}C cross polarization (CPMAS) measurements were performed with a Hartmann-Hahn match of 72 kHz with a ramped (90-100%) pulse on ^{1}H, a contact time of 3 ms, a recycle delay of 2 s, ^{1}H decoupling at 130 kHz using TPPM, and between 7k-50k scans (depending on sample). ^{1}H NMR was externally referenced to adamantane at 1.85 ppm,^{13}C NMR was externally referenced to adamantane at 38.5 ppm.

MALDI-TOF: The mass spectroscopic data were obtained at the Columbia University mass spectrometry facility using a Bruker ultrafleXtreme MALDI TOF/TOF with a frequency-tripled Nd:YAG laser (355 nm).

Electronic Absorption (UV-vis): Absorption spectra were obtained on a Shimadzu UV 1800 UV/vis spectrophotometer.

Visible-Light Flow Photocyclization: The flow reactor was a home-built reactor consisting of a peristaltic pump (Masterflex L/S PTFE-Tubing Pump System; 3 to 300 rpm, 90 to 260 VAC; Item# UX-77912-10), FEP tubing (Chemfluor FEP tubing), and 17,500 lumen LED cornbulb lamps (EverWatt, EWIP64CB150WE39NB24, 150 W). The tubing was wrapped around the LED bulbs to provide the reaction surface. No heating or cooling was applied, and a thermometer touched to the tubing during a reaction read ~55-65 ^{o}C. See supplementary reference 2 for related publication using an identical setup.[2]

Atomic Force Microscopy: Samples were prepared by spincoating soluble fractions of material in chlorobenzene onto HOPG. AFM maps were collected using a Bruker Dimension FastScan AFM. Histograms were generated by manually tracing visible ribbons using ImageJ.

Thermogravimetric Analysis: Measurements were performed at the Columbia University Shared Materials Characterization Lab using a TA Instruments Q500 TGA under constant flow of N_2. Approximately 5 mg of sample mass was loaded.

Cyclic Voltammetry: Measurements were recorded on a CH166 electrochemical workstation. 3 mL glass cells were used to contain 0.1 M tetrabutylammonium hexafluorophosphate ($TBAPF_6$) in dichloromethane as the supporting electrolyte. An Ag/AgCl electrode was used as the reference electrode, and voltammograms were then manually referenced to the known ferrocene/ferrocenium couple.

Procedures and Characterization:

Scheme S1: Synthesis of **cGNR-unc** (**1**)

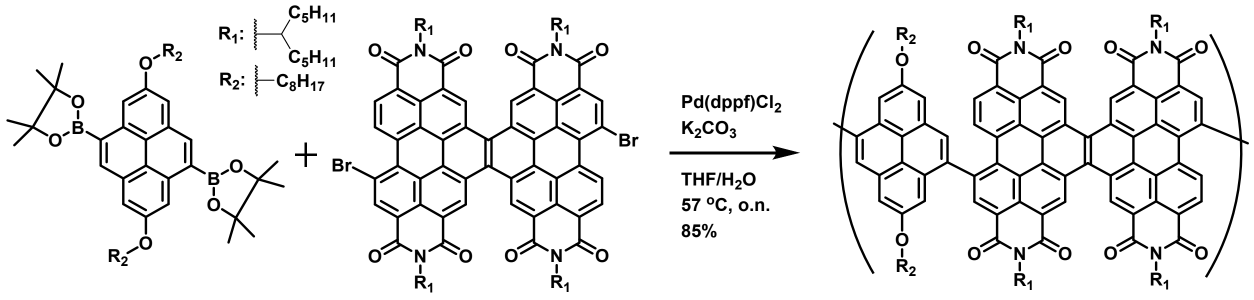

cGNR-unc (**1**):

A dry 3 mL vial was charged with a stirbar, diborylated-dioctyloxo-pyrene (90.2 mg, 0.127 mmol, 1 eq.), hPDI2-Br_2 (200 mg, 0.127 mmol, 1 eq.), [1,1′-bis(diphenylphosphino)ferrocene]dichloropalladium(II) (9.30 mg, 0.0127 mmol, 0.1 eq.), and potassium carbonate (134 mg, 1.02 mmol, 8 eq.). The charged vial was capped with a rubber septum, and subsequently evacuated and backfilled with nitrogen. Degassed water (0.2 mL) and degassed tetrahydrofuran (2.3 mL) were syringed into the vial. The mixture was then heated to 57 °C and stirred overnight (approx. 16 hr). The solution was then cooled to room temperature and diluted with water and chloroform. The organic fraction of the mixture was then filtered through Celite and washed with water, methanol, hexanes, and chloroform. The solution was then removed under reduced vacuum to yield a dark magenta solid (200 mg; 85% yield).

IR (ATR) [cm^{-1}]: 2956, 2916, 2850, 1700, 1660, 1587, 1463, 1406, 1325, 1244, 1186, 813, 720.

Scheme S2: Synthesis of **cGNR** (**2**):

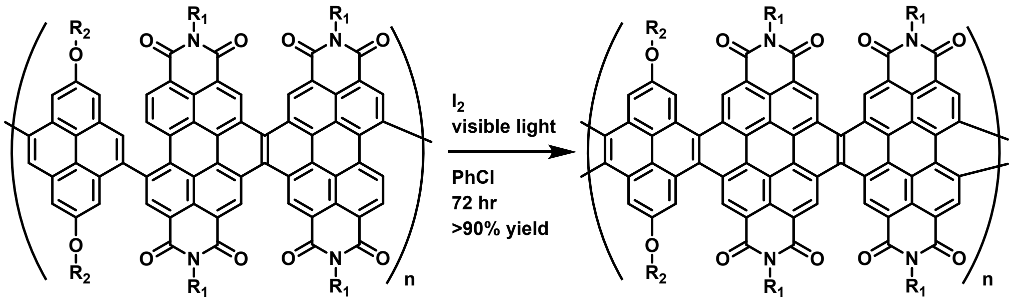

cGNR (**2**):

In a 100 mL round bottom flask, the uncyclized **1** (**cGNR-unc**) (100 mg) and iodine (25 mg) were dissolved in chlorobenzene (65 mL). The mixture was stirred for 15 minutes and then irradiated for 72 hr with visible light using the home-built flow reactor described above. The solvent was then removed under vacuum and the resulting solid was suspended in methanol and loaded onto a Celite plug. The solid was washed with methanol, hexanes, and acetonitrile and then dissolved in chloroform. The solvent was then removed under vacuum to give cyclized **2** (**cGNR**) as a dark green powder (90 mg). The resulting dark green solution was dried, and the solid mixture was separated using a Soxhlet extractor with methanol, hexanes, dichloromethane, and chloroform, consecutively. The resulting moderate-molecular weight **2'** (**cGNR'**) was contained within the dichloromethane fraction (32 mg), and the high-molecular weight **2** (**cGNR**) was contained within the chloroform fraction (52 mg). Total product recovered: 84 mg.

^{1}H NMR (500 MHz, $CDCl_3$, 323 K): δ 11.36 (bm, 16H), 9.37 (bm, 4H), 5.67 (bm, 8H), 4.85 (bm, 4H).

IR (ATR) [cm^{-1}]: 2955, 2920, 2852, 1703, 1661, 1596, 1442, 1412, 1306, 1236, 1183, 1124, 1067, 922, 812, 760, 722.

^{1}H and ^{13}C NMR Spectra:

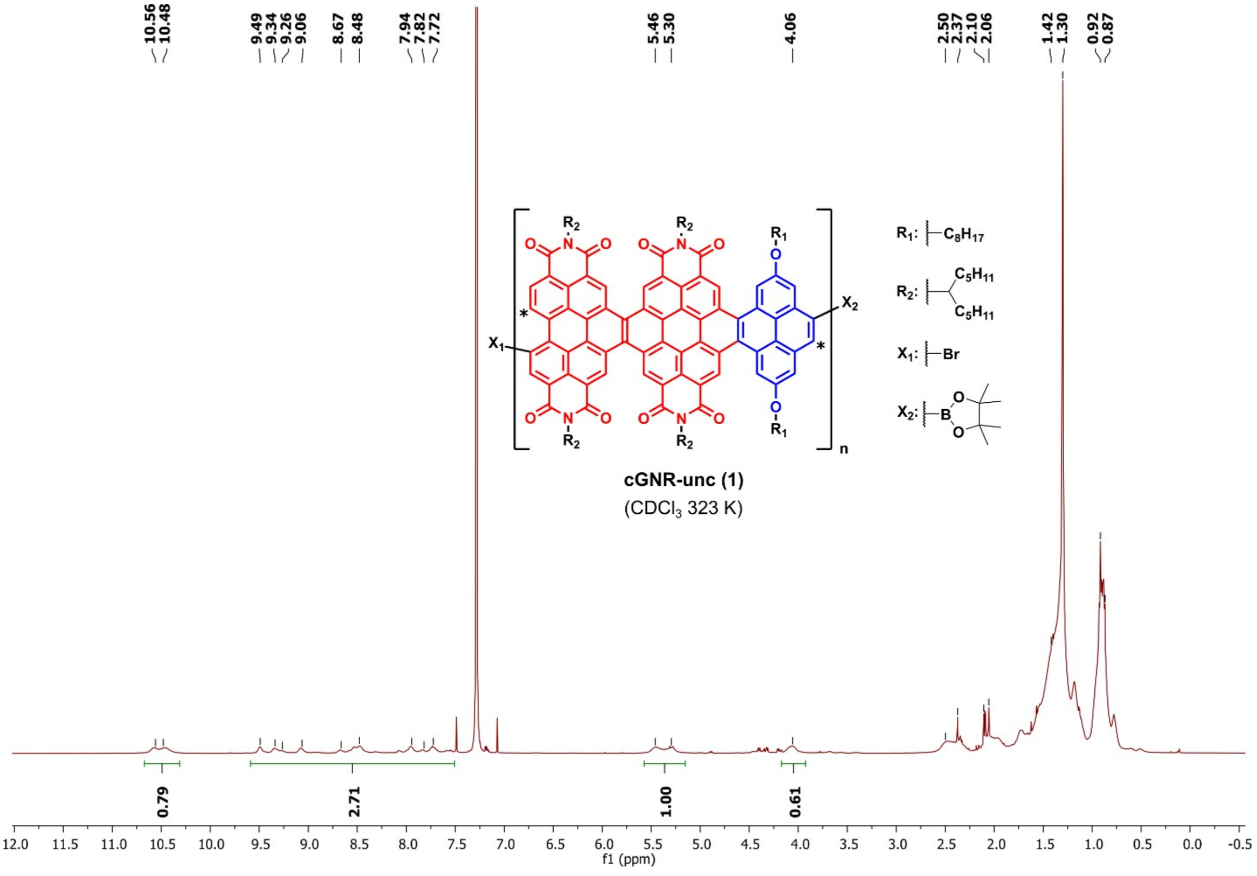

Figure S1. ^{1}H NMR spectrum of **1** in $CDCl_3$. Note the presence of broad signals in the δ 7.4-9.0 region, indicating the uncyclized state of the material.

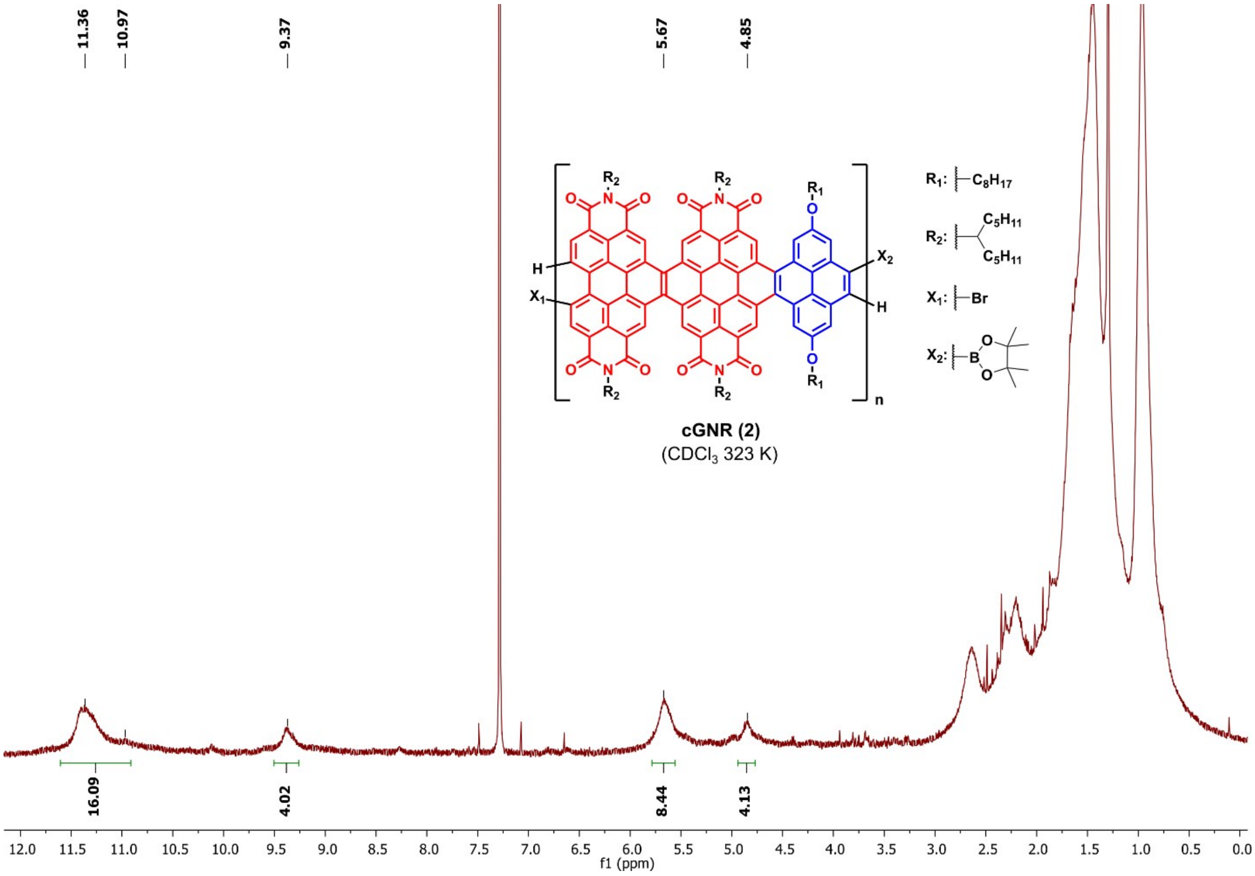

Figure S2. ^{1}H NMR spectrum of **2** in $CDCl_3$. Note the disappearance of broad resonances in the δ 7.4-9.0 region, indicating cyclization. This spectrum compares closely with the previously reported single molecule.[2]

Gel Permeation Chromatography:

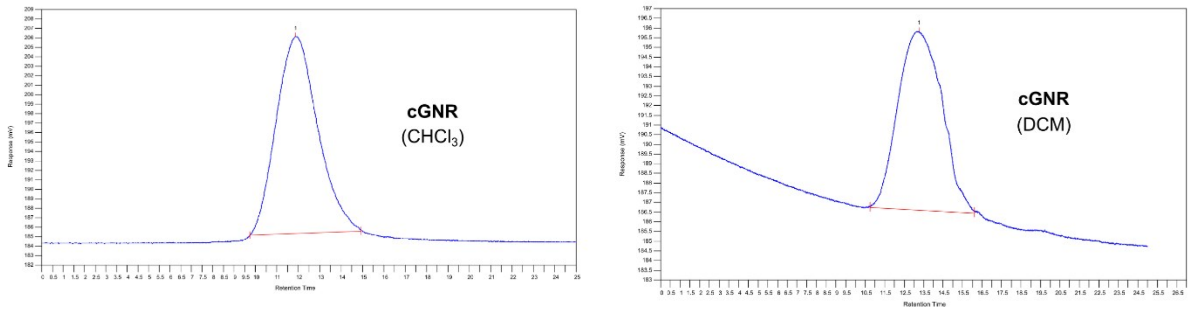

Figure S3. Gel permeation chromatography (GPC) traces for $CHCl_3$ (left) and DCM (right) polymer fractions. GPC calibrated to polystyrene SEC conditions. M_n and M_w observed for the **cGNR** species is approximate, and best corroborated by physical observation of provided AFM images below and within the maintext.

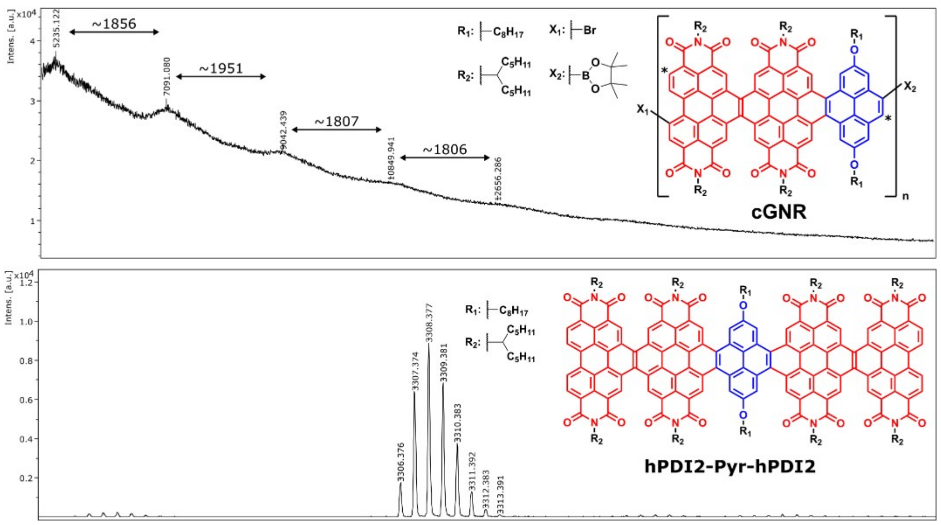

Figure S4. MALDI-TOF (positive mode) of the contorted GNR (top frame) and the single-molecule variant previously reported (bottom frame).[2] More accurate masses proved to be prohibitively difficult. The average spacing for the top panel is ~1855 Daltons, which roughly corresponds to the expected unit weight of 1868.396 Daltons.

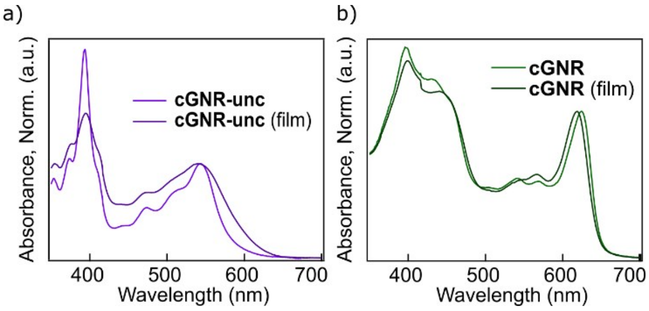

Figure S5. Electronic absorption spectra of (a) the uncyclized species **cGNR-unc** (**1**) in both solution and film, and (b) the cyclized species **cGNR** (**2**) in both solution and film.

Gas Chromatography-Mass Spectrometry:

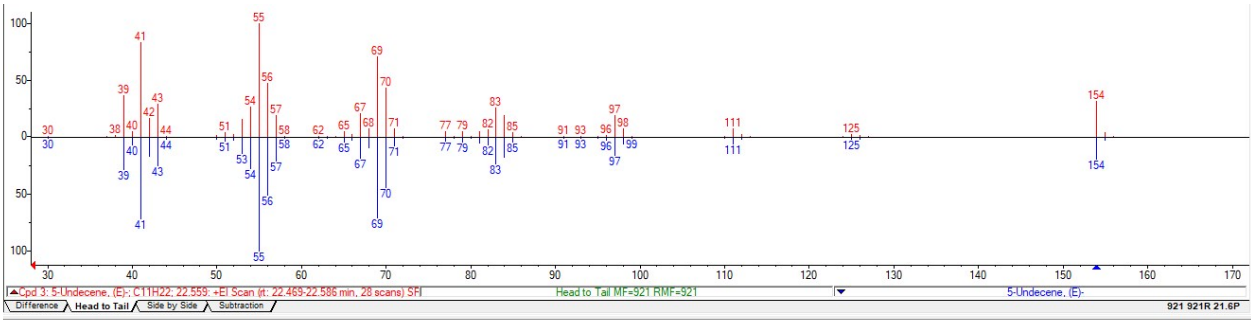

Figure S6. Gas chromatography-mass spectrometry (GC-MS) library reference for 5-undecene (blue) and observed sample of removed alkyl chains (red).

IR Fingerprint Region:

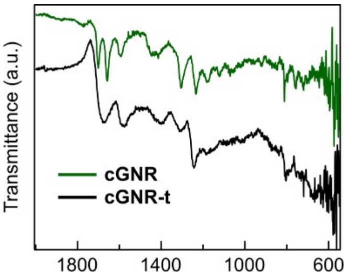

Figure S7. Fingerprint region in the IR spectra of both cyclized **cGNR** (**2**) and thermolyzed **cGNR-t** (**3**). Graphene nanoribbon character is maintained following thermolysis.

Raman Spectroscopy:

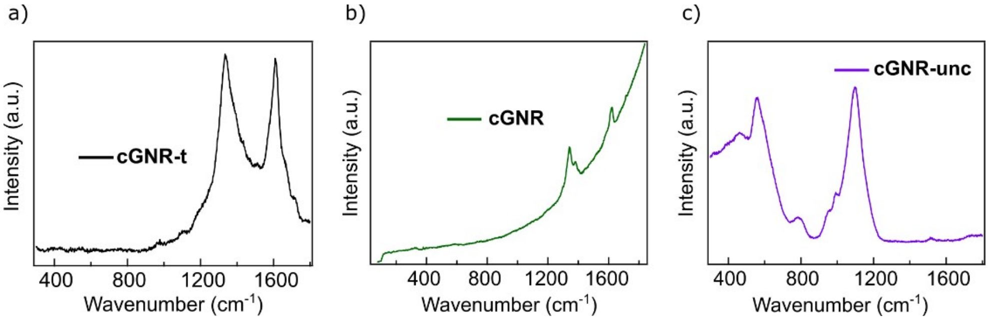

Figure S8. a) Raman spectrum (powder, 532 nm excitation wavelength) of cyclized, thermolyzed ribbons **cGNR-t** (**3**). Note the retention of the characteristic GNR D-band (~1334 cm^{-1}) and G-band (~1608 cm^{-1}). This indicates that the GNR structure is preserved following thermolysis. b) Raman spectrum (film, 532 nm excitation wavelength) of cyclized, unthermolyzed ribbons **cGNR** (**2**). Note the presence of the characteristic GNR D-band (~1334 cm^{-1}) and G-band (~1608 cm^{-1}). This indicates that the GNR structure is formed following photocyclization. c) Raman spectrum (film, 532 nm excitation wavelength) of uncyclized, unthermolyzed ribbons **cGNR-unc** (**1**). Note the lack of characteristic graphene nanoribbon (GNR) D- and G- bands (~1330 cm^{-1} and ~1600 cm^{-1}, respectively).

GNRs + Dopant Current Measurements:

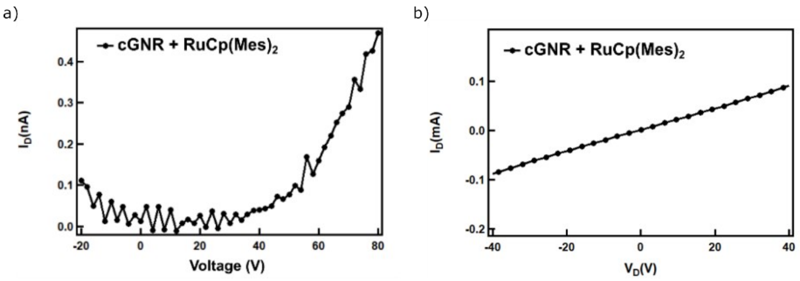

Figure S9. a) Transfer curve for thin film transistors fabricated with **cGNR** doped by ruthenium pentamethylcyclopentadienyl mesitylene dimer ((RuCp*mes)$_2$).[3] This device was prepared by spincoating with 10 mg/mL **cGNR** solution mixture with 1% (RuCp*mes)$_2$ directly on the Si/SiO$_2$ chip pre-patterned with Au electrodes (channel length = 20 µm, channel width = 105 µm). b) Current-voltage curve of **cGNR-t** doped with 1% (RuCp*mes)$_2$. We firstly prepared the **cGNR** thin film transistor through spin coating 10 mg/mL **cGNR** solution on the Si/SiO$_2$ chip pre-patterned with Au electrodes ((channel length = 20 µm, channel width = 105 µm). Then we submitted the chip for thermolyzing. Finally, we spin coated 0.1mg/mL (RuCp*mes)$_2$ solution on the chip. This yields a current of 5.5 mS/m.

References

(1) Zhong, Y.; Kumar, B.; Oh, S.; Trinh, M. T.; Wu, Y.; Elbert, K.; Li, P.; Zhu, X.; Xiao, S.; Ng, F.; Steigerwald, M. L.; Nuckolls, C., *J. Am. Chem. Soc.* **2014,** *136*, 8122.
(2) Sisto, T.J.; Zhong, Y.; Zhang, B.; Trinh, M. T.; Miyata, K.; Zhong, X.; Zhu, X.-Y.; Steigerwald, M. L.; Ng, F.; Nuckolls, C., *J. Am. Chem. Soc.* **2017**, *139*, 5648.
(3) Avila, J.; La-Placa, M. G.; Longhi, E.; Sessolo, M.; Barlow, S.; Marder, S. R.; Bolink, H. J.; *J. Mater. Chem. A*. **2019**, *7,* 25796.
